# Possibilities of Integrated Fabrication of Insulation Systems in Electric Drives by Injection Molding of Thermosets

**DOI:** 10.3390/polym14245352

**Published:** 2022-12-07

**Authors:** Uta Rösel, Maximilian Kneidl, Dietmar Drummer, Jörg Franke

**Affiliations:** 1 Institute of Polymer Technology, Friedrich-Alexander-Universität Erlangen-Nürnberg, 91058 Erlangen, Germany; 2 Institute for Factory Automation and Production Systems, Friedrich-Alexander-Universität Erlangen-Nürnberg, 91058 Erlangen, Germany

**Keywords:** thermoset, injection molding, insulation system

## Abstract

Due to the increasing demand for electro mobility and specifically for electrified vehicles, the demand for electric drive technology is expanding significantly with changing requirements in terms of the process and the application. The electrical insulation system of the stator is an essential part of the fabrication process with a high impact on the application properties. Due to limitations—for example, in terms of suitable materials for the stator insulation—a new technology of integrated fabrication by injection molding of thermosets has been founded. In this study, two epoxy (EP) types with different fillers were investigated to prove their suitability in terms of the material properties in the fabrication process and the application. A general realization of the integrated fabrication of insulation systems in electrical engineering by injection molding was proved. Further, the differences regarding the suitability of the two materials are portrayed. It was demonstrated that mainly the filler material influences the fabrication process and the properties in the application, leading to differing suitability in terms of the EP 3162 EMG within the fabrication process and in terms of XW 6640-1 within the application properties of the thermal conductivity and the thermal linear expansion. It was further shown that the filler within the material system is required to increase the thermal conductivity needed for the application. The inclusion of the filler influences the reaction kinetics and the viscosity behavior. A fabrication of the material with fillers is however still possible.

## 1. Introduction

The increasing demand for electro mobility especially in terms of electrified vehicles and powertrains is expanding the development of electric drive technology significantly. Further, the requirements not only in terms of the product but also in terms of the fabrication process is changing. To reduce the manufacturing costs and achieve economic efficiency, a high degree of automation in the production with low rejection rates is required to face the increasing demand. Concerning the product, a high power density and optimum efficiency of the electric drive unit must be reached while realizing low weight and small installation space [1]. The stator insulation system determines the power of the motor with respect to the entire life cycle of the application due to thermal and electrical restrictions [2]. Therefore, the electrical insulation system reveals a crucial position within the development of electro mobility applications.

The classic stator insulation system consists of two groups of insulation—the primary insulation covering the conductors and the secondary insulation in the slot and of the full stator by impregnation. The insulation of the conductors provides the basic electrical insulation to avoid electrical flashovers between wires, which would lead to a reduction in performance. Common insulation materials of the conductors are varnishes consisting of polyamide-imide (PAI) or foils of polyimide (PI). The foils are wrapped several times around the conductor, to ensure sufficient insulation of multiple layers. To meet the increasing requirements in applications within the automotive or aerospace industries, high temperature polymers such as polyether ether ketone (PEEK) or polyphenylene sulfide (PPS) are used [3]. The slot ground insulation increases the lagging of the conductors against the laminated core of the stator further and protects the primary insulation from damage in the process of inserting the coils into the laminated core. With that, flat insulating materials such as foils out of polyester imides or aramides as well as inorganic materials such as mica are used within a multi-layer structure [2]. In the final insulation step, the windings are fixed in their position by impregnation using an epoxy (EP) or unsaturated polyester resin for example. This step requires a significant proportion of the entire production time of the stator and ensures both a mechanical and chemical protection of the windings as well as a dissipation of heat, which is generated in the conductor during application [4]. The methods of the final insulation step can be categorized as impregnating and casting with processing under vacuum, atmosphere, or overpressure, as well as a combination of them. The casting includes a full encapsulation of the stator, where the windings with the winding heads are completely enclosed with resin. The quality of the insulation system is higher compared to the impregnated systems because of fewer defects. However, the processing time as well as the material consumption is significantly higher.

The impregnation of the stator windings is realized by liquid insulating resin followed by a thermal curing process. The method is subdivided into dip, roller, and trickle impregnation. Within the dip and roller impregnation, the preheated stator is placed into a soaking tank filled with impregnating resin, where the resin infiltrates the winding. Due to the high temperature of the preheated stator, the resin slowly reaches the gelation point and cures. Afterwards, the excess material has to be removed. Depending on the resin, the curing process can be accelerated by applying additional current to the windings or by using a UV lamp [5]. In the trickle impregnation, the resin is poured onto the winding heads by an individual nozzle. The infiltration takes place due to the capillary effect, which forces the resin into the bottom of the slots and thus impregnates the entire stator with significantly less resin used compared to dip- or roller-based processes. However, the methods of soaking reveal a higher possibility of defects, such as trapped air, compared to the casting processes, which can lead to a weakening of the insulation strength of the system [5]. The different methods of the impregnation reveal several challenges, to realize an efficient technology which can be used in large-scale production. So far, the main limitations are long cycle times of the semi-finished products and the curing of the resin, and lack of modification of the material behavior in terms of the thermal conductivity and mechanical strength, which are both important for the application and lack of process control and sufficient insulation quality.

The production of the stator is divided into the production of the laminated core, the slot base insulation, the winding of the coils, the impregnation of the windings, and the contact of the conductors [6]. For applications in the automotive industry, the impregnation of the stator is usually carried out using trickle impregnation, which results in long process times due to the curing, low reproducibility of the samples, and the low thermal conductivity of the resins as low viscosities have to be reached [1]. The limitations of the impregnation processes can be expanded by a different method using thermoset injection molding. Within this process, the thermoset resin is placed into a low-temperature unit and plasticized as well as homogenized using a screw. The material is injected into the cavity, which causes a temperature change. The viscosity of the thermoset drops abruptly due to the increase of the temperature before the overlapping of the irreversible crosslinking reaction causes the viscosity to increase again [7]. This process allows application of pressure during injection and curing due to the closed mold, which significantly reduces defects, such as trapped air and leads to a reproducible component weight [8]. Further, the process of injection molding enables short cycle times, improved handling conditions of the material, and a modification of the material with respect to the demand of the application [1]. The material used in the injection molding process is a granulated thermoset based on a premixed resin hardener system with up to 85 mass% of fillers. These fillers allow a wide range of modifications of the material behavior and the properties achieved in the application. For example, the thermal conductivity of thermosets can be increased from 0.2 $\frac{W}{m\cdot K}$ to over 2.5 $\frac{W}{m\cdot K}$ [8].

The basic requirement for using thermoset injection molding for the insulation of stators is the complete impregnation of the electrical conductors by the thermoset. Impregnation is defined as the flow of a liquid through a porous medium [9]. The porous medium in the stator is covered by the windings and the slots between the conductors. With reference to [10], in the two-dimensional perspective the flow path of the liquid and thus, the impregnability is proportional to the viscosity of the liquid, the permeability of the porous medium, the impregnation time, and the processing pressure. These impact factors can be adaptable to the injection molding process with the exception of the viscosity of the liquid, since a thermoset molding compound is not a Newtonian fluid, as assumed by [10]. The complete impregnation of conductor alike structures with highly filled thermosets in the injection molding process was shown on carbon rovings by [11]. It was demonstrated that several process parameters directly influence the impregnation quality. An increasing tool temperature, for example, reduces the viscosity leading to an improvement in the impregnation and realizing complete insulation, analogous to [12]. The general use of the injection molding process to fabricate a stator has already been realized to some extent [12, 13]. However, only small dimensions of the stator with short flow paths or stator segments have been implemented so far.

The aim of this paper is to investigate the suitability of epoxy-based resins for the application of insulation of stators by injection molding. The main material requirements are summarized in Table 1. The target values of these parameters are based on the standard process conditions as well as the application requirements. For example, the low viscosity is a precondition for long flow paths as this allows not only full insulation of the wires due to a high fluidity, but further a longer time with that flow path in the presence of curing parameters. The requirement for the reaction kinetics is rather complex and is therefore here not further defined. The evaluation of the reaction kinetics is presented within Section 3. The high thermal conductivity is needed with respect to the application in stator systems, as temperature rise during the usage of the stator must be dissipated easily to reduce the thermal input in the system and increase the durability. The low thermal linear expansion is based on the stator assembly, which is based on different materials such as the copper in the wire or the metal sheets in the statorette. By reaching a low thermal linear expansion, higher eligibility between the different materials is given, which increases the lifetime again. The target values in terms of the average of the partial discharge and the partial discharge inception/extinction voltage go along with the application and indicate sufficient insulation of the wires. The paper investigated two commonly used epoxy resins and reveals their general material properties as well as the fabrication of stator segments with a long flow path, to evaluate their suitability in terms of the fabrication process and the application.

## 2. Materials and Methods

### 2.1. Material

The experiments were conducted using two commercial types of epoxy resin (EP)—the type EP 3162 EMG (Raschig GmbH, Ludwigshafen, Germany) and the type XW 6640-1 (Duresco GmbH, Witterswil, Swizerland), which differ in terms of the anorganic filler material, but are both based on bisphenol-A. Both materials are a premixed grey-black granulate with resin, hardener, catalyst, and some carbon black pigments. The exact composition of the mixture including the filler type and grade is a business secret of Raschig GmbH or Duresco GmbH and therefore confidential. Characterization of the pure filler systems is unfortunately not possible due to the business secret. Table 2 reveals the important properties of the used material based on our own measurements. Further characterization of the material is revealed in the datasheets of the materials [14,15]. To define the properties needed for fabrication of the materials, a wide characterization of the materials was conducted in this study.

**Figure 1:**
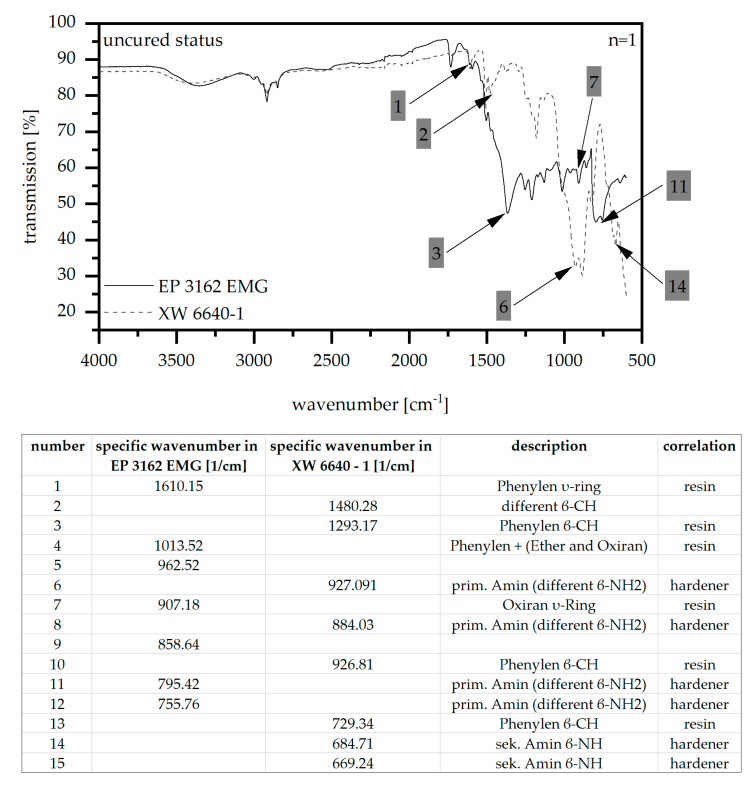
IR-spectra of EP 3162 EMG and XW 6640-1.

Figure 1 depicts the IR spectrum of the ATR method of the two types of epoxy resin with the main difference in the bands highlighted. The different bands are related to possible elements according to [16]. As it can be seen from the IR-spectrum there is a significant difference in the components and the proportion of resin and hardener in the two epoxy types with respect to the different bands. However, the IR spectrum is similar for the range of wavenumbers between 2.500 and 4.000 cm^−1^. The difference in bands of the two epoxy types is also a clue with respect to the filler type chosen. Taking the NIST Standard reference database into account, the characteristic peak (position 3) of EP 3162 EMG suggests the use of boron nitride, whereas the characteristic peak (position 6) of XW 6640-1 depicts the use of aluminum oxide.

Both filler types are chosen mainly with respect to their high thermal conductivity, which is one of the demands in terms of the utilization of resins within the integrated fabrication of insulation systems in electrical engineering. Further, the filler in EP 3162 EMG has a high electric insulation property, which serves another demand in terms of the application. While the filler itself could not be characterized due to the business secret, the material behavior was compared to a pure epoxy resin type EP 3681 E (Raschig GmbH, Ludwigshafen, Germany), which is based on the same resin, hardener, and catalyst without fillers. The density of this type is 1.225 g/cm^3^.

### 2.2. Fabrication of the Test Specimens

**Figure 2:**
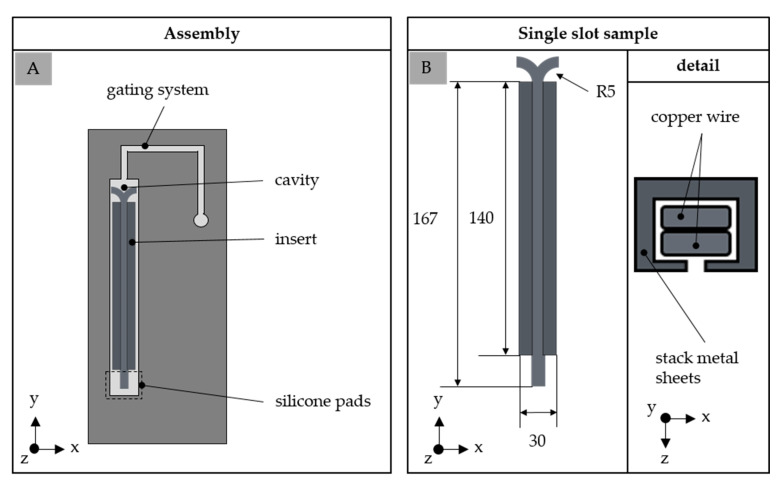
Schematic assembly of the tool to fabricate the single slot sample (**A**) and detailed dimension of the sample (**B**).

The test samples were produced pressure controlled by a Krauss Maffei KM 80-380 CX DUR/03 injection molding machine (KraussMaffei Group, Munich, Germany) with a screw diameter of 30 mm. Two types of test samples were produced; in terms of the main material characterization, plates with the dimension of 60 × 60 × 2 [mm^3^] were fabricated in a dual cavity to prepare the test samples needed for the different characterizations. Further, test samples—so-called single slot samples—were produced to evaluate the suitability of the material with respect to the application. The assembly of the tool is shown in Figure 2, where one stack of metal sheets together with two wires are inserted into the tool before the injection molding process starts. The cavity is sealed on the opposite side of the gate using silicone pads to ensure accessible terminals.

The processing parameters were set as shown in Table 3. Due to different test sample volumes, the processing parameters for the two types of samples differ slightly in terms of the mold temperature and the holding pressure. However, the main parameters were kept constant for both samples.

### 2.3. Characterization

To evaluate the suitability of the material in terms of the demand of the application the material was characterized with respect to the injection molding process, the fabrication in the test sample, and the resulting properties in the application. For each characterization a material recommendation is given respectively.

#### 2.3.1. Specific Heat Capacity c According to ISO 11357-4

To evaluate the material impact, specifically due to the fillers, on the temperature control and the flow and curing process, the specific heat capacity c was determined at 25 °C using the C80 calorimeter (type: 3D-Calvet calorimeter; TA Instruments, New Castle, DE, USA). Although the specific heat capacity c is temperature dependent, the measured parameters are assumed to be valid in terms of evaluating the impact of the material on the curing.

#### 2.3.2. Thermal Conductivity a According to DIN EN 821

To analyze the thermal conductivity a in terms of the process as well as the application properties, plate-like samples of the dimension 12.7 × 12.7 [mm^2^] were prepared from the middle of the test samples of the plate. The measurements were carried out for the temperature sets of 23, 80, 120, and 160 °C, to consider the temperature dependence within the analysis. Three samples per temperature set were examined using a Nanofash (type: LFA 447; Netzsch-Group, Selb, Germany).

#### 2.3.3. Thermal Linear Expansion ΔL According to ISO 7991

The thermal linear expansion ΔL was determined after the preparation of a sample with the dimension of 4 × 4 [mm^2^] from the test sample of the plate, using a thermomechanical analyzer (type: TMA 450; TA instruments, New Castle, DE, USA). The analysis was set between −20 and 200 °C with a heating rate of 3 °C per minute. The thermal linear expansion ΔL has mainly an impact on the application properties.

#### 2.3.4. Differential Scanning Calorimetry (DSC) According to ISO 11357

To investigate the temperature dependent reaction kinetics in terms of the process conditions of the material, a differential scanning calorimetry (DSC Q100; TA Instruments, New Castle, DE, USA) was used. Samples of about 5 mg were placed in DSC aluminum pans and heated with a constant rate of 10 °C per minute from 0 °C to 240 °C. The experiments were conducted in a nitrogen atmosphere with a flow rate of 50 mL per minute. To characterize the curing process, the specific enthalpy ΔH_ges; 1_ and the peak temperature T_peak_ were determined. Further, the reaction turnover α was calculated considering Equation (1) where ΔH_j_ is the specific enthalpy at the temperature T_j_ and ΔH_ges;1_ is the total specific enthalpy in the first heating cycle [7].

(1) $$\alpha =\left(\frac{\Delta H_{j}}{\Delta H_{ges;1}}\right)*100\;\left[\%\right]$$

#### 2.3.5. Determination of the Viscosity Using a Rotational Viscometer According to DIN EN 6043

To characterize the viscosity, as one of the most important processing conditions in terms of thermosets, a rotational viscometer (Discovery Hybrid Rheometer 2; TA Instruments, New Castle, DE, USA) was used with DIN EN 6043. The viscosity was determined with respect to increasing temperature (dynamic behavior) and to time dependence (isothermal). The assembly was based on two plates with a shearing load rotating with a constant frequency of 1 Hz. In the case of the dynamic measurements, the temperature range was set between 90 °C and 200 °C with a constant heat rate of 5 °C per minute. The minimum of the viscosity η_min_ and the corresponding temperature Tη_min_ were analyzed.

The isothermal measurements started at a certain temperature, which was held constant, and the change of the viscosity depending on the time was determined. This isothermal plateau was set at a temperature of η_min_, which was 120 °C in the case of EP 3162 EMG and 110 °C for XW 6640-1, and was further increased in steps of 20 °C up to 180 °C ongoing from 120 °C. The route of the viscosity η relative to the time with respect to a constant temperature follows an s slope. The time t_pot_ between the beginning of the calculation and the turning point—so-called pot life–was analyzed.

#### 2.3.6. Microscopy

To analyze the position of the wire within the stack and the insulation of the wire due to the injection molding process, small strips of the single slot sample were removed using a water-cooled saw with minimal temperature input. The strips were taken from positions near and far away from the gate, to evaluate the change of the position of the wires and the polymer along the flow path. The strip samples were embedded in cold-curing epoxy resin (type: Epofix; Struers GmbH, Ottensoos, Germany) and polished.

Afterwards, the samples were characterized by a stereo microscope (type: Axio Zoom.V16; Carl Zeiss AG, Oberkochen, Germany) to create an overview image and further characterized by a reflected light microscope (type: Axio Imager.M2; Carl Zeiss AG, Oberkochen, Germany) to create images in more detail.

#### 2.3.7. Average of Partial Discharge and Partial Discharge Inception/Extinction Voltage

**Figure 3:**
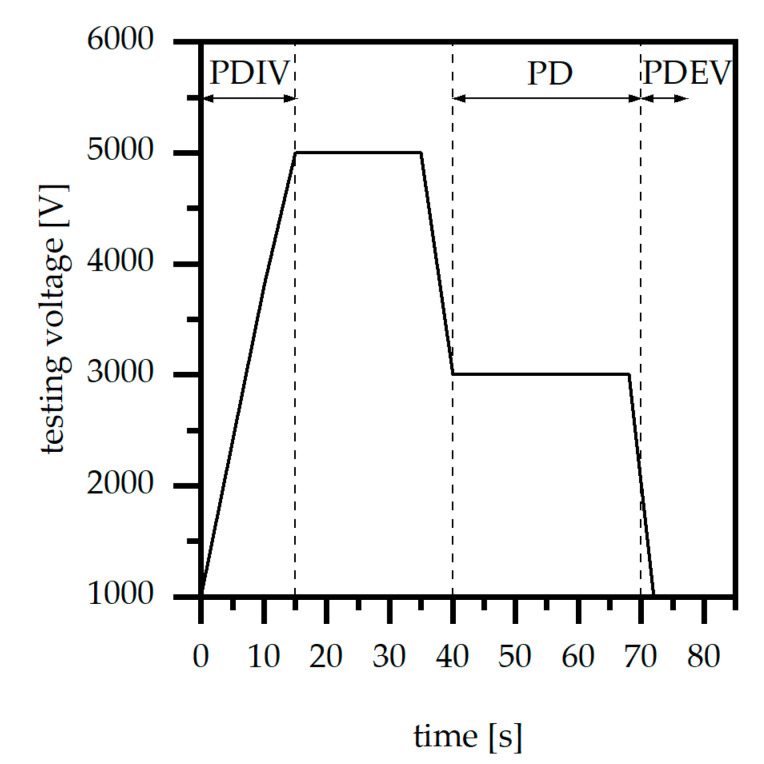
Testing voltage for the measurement of the average of partial discharge (PD) and the partial discharge inception (PDIV) as well as extinction voltage (PDEV).

The evaluation of the partial discharge (PD) and the partial discharge inception (PDIV) as well as the extinction voltage (PDEV) were realized using a test equipment with two parallel electrodes, which is defined by IEC 60243-1, and a measuring system within a Faraday cage according to IEC 60270 (type: Omicron MPD 600; Omicron electronics GmbH, Klaus, Austria). The test samples were provided by the material supplier and had the dimension of 150 × 150 × 4 [mm^2^] in terms of EP 3162 EMG as well as 100 × 100 × 4 [mm^3^] for XW 6640-1. As the thickness of the plates is the only dimension, which impacts the measurement, the different cross sections of the plates in terms of the two materials do not affect the testing. After clamping the test samples within the test setting, the values of the partial discharge level, the partial discharge inception, and the extinction voltage were defined using the testing profile as shown in Figure 3. The testing time for the partial discharge inception voltage (PDIV) was defined with 15 s, and for the partial discharge level (PD) for 30 s. Further, the voltage incline was 100 V per second. The experiments were held at a room temperature of 20 °C with a humidity of 50%.

## 3. Results

### 3.1. Specific Heat Capacity c According to ISO 11357-4

The specific heat capacity c reaches 0.997 $\frac{J}{g\cdot {}^{{}^{\circ}}C}$ in terms of the material EP 3162 EMG and 0.899 $\frac{J}{g\cdot {}^{{}^{\circ}}C}$ in terms of XW 6640-1. For the pure epoxy resin c reaches 1.616 $\frac{J}{g\cdot {}^{{}^{\circ}}C}$, which shows that the fillers implemented in the resin reduce the specific heat capacity c. As c should reach a high value with respect to the application, as shown in Table 1, EP 3162 EMG reveals slightly higher suitability in the application, although the difference between the two materials is less.

### 3.2. Thermal Conductivity a According to DIN EN 821

**Figure 4:**
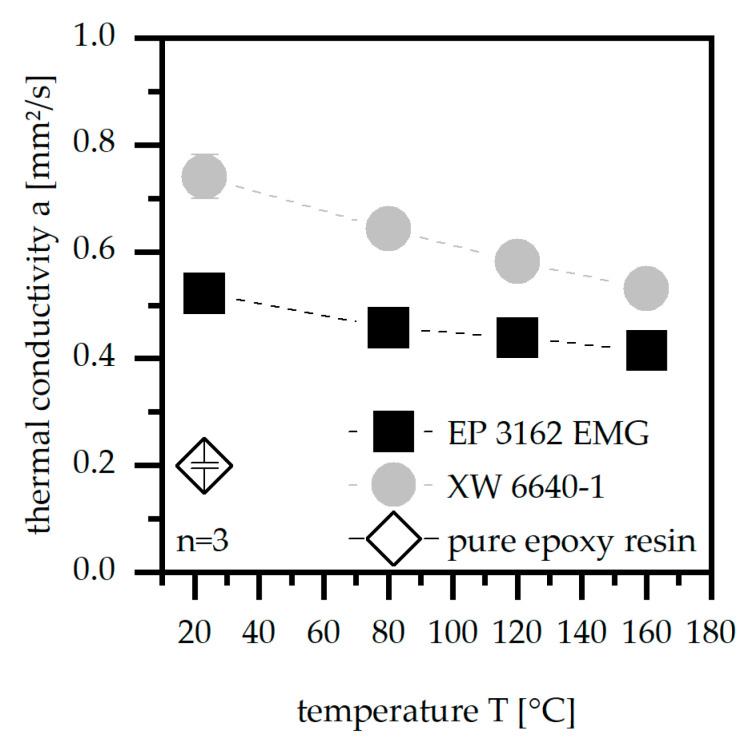
Thermal conductivity a for the temperature sets of 23, 80, 120, and 160 °C compared for two EP types and the pure epoxy resin.

The thermal conductivity a, as shown in Figure 4, is reduced with increasing temperature T independent of the material. Further, the standard deviation is higher in terms of the temperature of 23 °C for both materials compared to the other temperature sets. The material XW 6640-1 reaches about 40% higher values in terms of the low temperature sets. The difference in the values between the two materials is reduced to 25% higher values for higher temperatures. Nevertheless, the material XW 6640-1 reaches higher values for a and reveals therefore higher suitability in terms of Table 1. It can be also seen that the fillers in both materials increase a compared to the pure epoxy resin, which reveals a significant lower value for a at a temperature of 23 °C.

### 3.3. Thermal Linear Expansion ΔL According to ISO 7991

**Figure 5:**
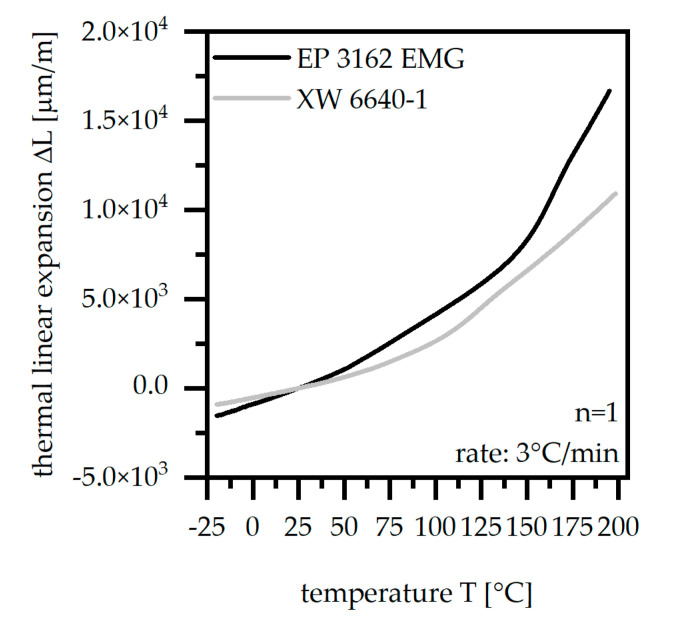
Thermal linear expansion in the temperature range of –20 up to 200 °C compared for two EP types.

The thermal linear expansion ΔL increases between −20 °C and 200 °C for both materials with different gradients. For low temperatures up to 60 °C, the linear route of ΔL reveals a higher gradient in terms of EP 3162 EMG, which starts at a lower value at −20 °C compared to XW 6640-1. Above 60 °C, the thermal linear expansion ΔL increases much faster in terms of EP 3162 EMG, which leads to inhomogeneous behavior over the whole temperature range. The route of the thermal linear expansion ΔL compared for both materials can be seen in Figure 5. With respect to the material requirements and Table 1, XW 6640-1 complies with the demand with a higher amount due to the lower ΔL and a more homogeneous route compared to EP 3162 EMG.

In comparison to the thermoset material, the stack metal sheets reveal a thermal linear expansion ΔL of 28 $\frac{\mu m}{m}$ at 150 °C with only a small range over the temperature range.

### 3.4. Differential Scanning Calorimetry (DSC) According to ISO 11357

**Figure 6:**
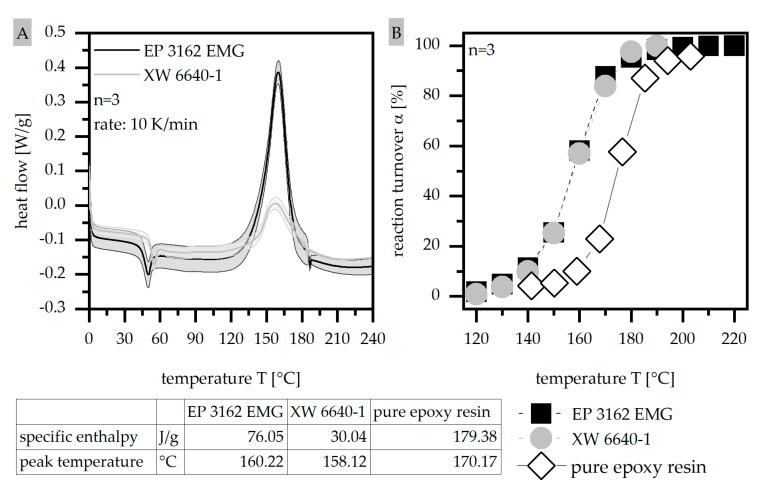
Route of DSC measurements with specific enthalpy ΔH_ges;1_ and peak temperature T_peak_ (**A**) as well as reaction turnover α (**B**) compared for two EP types and the pure epoxy resin.

The route of the DSC measurements as well as the specific enthalpy ΔH_ges;1_ and the peak temperature T_peak_ (A) together with the reaction turnover α (B) are shown in Figure 6 in comparison for the two different EP types of EP 3162 EMG and XW 6640-1 as well as the pure epoxy resin. The reaction kinetics of the two materials with fillers reveal different values in terms of the specific enthalpy ΔH_ges;1_ with only 50% needed in terms of XW 6640-1 compared to EP 3162 EMG, but similar behavior regarding the reaction turnover α and the peak temperature T_peak_. The reaction turnover α illustrates an s slope as expected. The level of the specific enthalpy ΔH_ges;1_ and the peak temperature T_peak_ is mainly influenced by the material in terms of the different heat capacity c. With that, XW 6640-1 requires less applied heat in the curing process. As the difference between the two materials within the reaction kinetics is only present in terms of the level of ΔH_g es;1_, a similar behavior during the fabrication regarding the process parameters is likely. However, EP 3162 EMG depicts a higher standard deviation, leading to more unstable process conditions. Compared to the pure epoxy resin, the specific enthalpy ΔH_g es;1_ is significantly reduced by the fillers as they do not participate within the hardening process. As the thermal conductivity a is increased by the filler systems, the reaction turnover α is shifted to lower temperatures in terms of the filler systems compared to the pure epoxy resin. With that, the presence of the fillers in the two material systems reduces the specific enthalpy ΔH_ges;1_ needed for the reaction and changes the temperature of the curing with respect to the reaction turnover α.

### 3.5. Determination of the Viscosity Using a Rotational Viscometer According to DIN EN 6043

**Figure 7:**
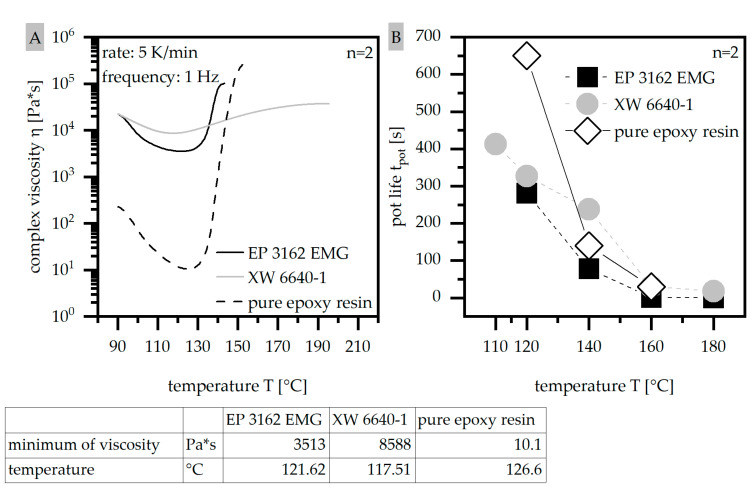
Route of the dynamic complex viscosity η with minimal complex viscosity η_min_ and corresponding temperature Tη_min_ (**A**) as well as pot life t_pot_ (**B**) compared for two EP types and the pure epoxy resin.

The route of the complex viscosity as dynamic behavior is shown in Figure 7A for the two different EP types. EP 3162 EMG reveals about half a decade lower viscosity relative to XW 6640-1 with a slightly higher temperature of the minimum of viscosity. After reaching the minimum of the viscosity, EP 3162 EMG increases over a small range of temperature and gains much higher values compared to XW 6640-1. Compared to the pure resin the minimum of the viscosity is increased in terms of the filler systems by at least three decades. Figure 7B further shows the pot life t_pot_ for both EP types, where XW 6640-1 reaches higher values compared to EP 6640-1. With respect to the pure epoxy resin the pot life t_pot_ is only increased for low temperatures. With increased temperature the difference between the material systems with and without filler is reduced. Overall, this results in a lower sensitivity of XW 6640-1 regarding the impact of time and temperature during the curing. As the difference in the values of t_pot_ between the two materials is low, the significantly lower viscosity in terms of EP 3162 EMG is the crucial factor to evaluate the viscosity behavior of the two EP types. Therefore, EP 3162 EMG is defined as more suitable in terms of the flow conditions, as the low viscosity is more likely to realize long flow paths. The higher pot life t_pot_ makes the material system more sensitive in terms of the impact of time and temperature. However, it can be used in terms of low fabrication times leading to cost reduction in the production process. The high increase of EP 3162 EMG after reaching the minimum of the viscosity refers to fast curing after reaching a certain temperature set-up. This can be used in terms of low fabrication times, however it has to be taken into account that this might also hinder the achievement of long flow paths. The low viscosity enhances the possibility of long flow paths, but fast curing reduces them.

### 3.6. Microscopy

**Figure 8:**
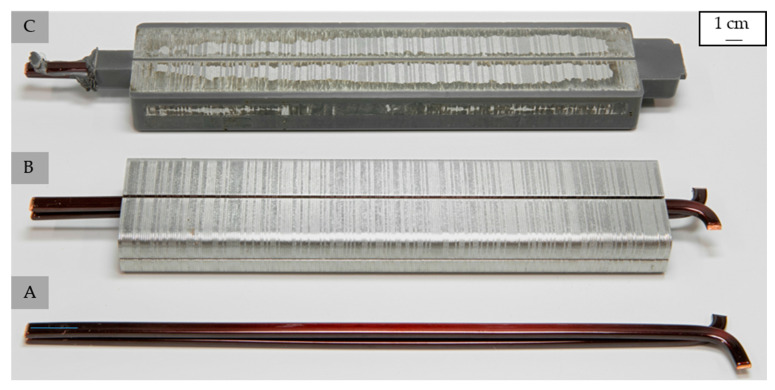
Assembly of the single slot sample with the copper wire (**A**), copper wires inserted into the stack metal sheets (**B**), and test sample after the injection molding process (**C**) [exemplary: EP 3162 EMG].

The assembly of the single slot sample is shown in Figure 8 with the copper wire itself (A), the copper wire inserted into the stack metal sheets (B), which functions as the inserts in the injection molding process, and the sample after fabrication (C). The sample reveals full filling of the cavity and the possibility of sealing through silicon pads, as the material load on the copper wire stops at a defined position.

**Figure 9:**
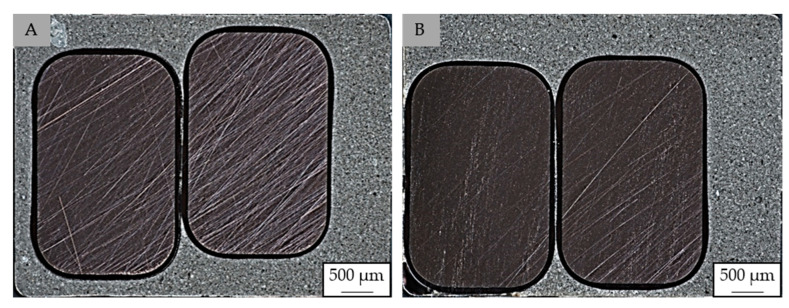
Position of the conductor in the stack metal sheet near the gate (**A**) and far away from the gate (**B**) [exemplary: EP 3162 EMG].

Further, Figure 9 depicts the position of the conductor in the cavity near the gate (A) and far away from the gate at the end of the flow path (B). It can be seen that the position of the wires changes along the flow path leading to a replacement of the conductor on the opposite side of the gating system. While the straight wire terminals are clamped near position B, the curved ends near the gating (A) remain unfixed. This change in the position might occur due to the forces of the injection process. It can be assumed that the optimization of the process parameter can reduce this movement. At this moment, full insulation of the conductor is yet not given at the end of the flow path.

**Figure 10:**
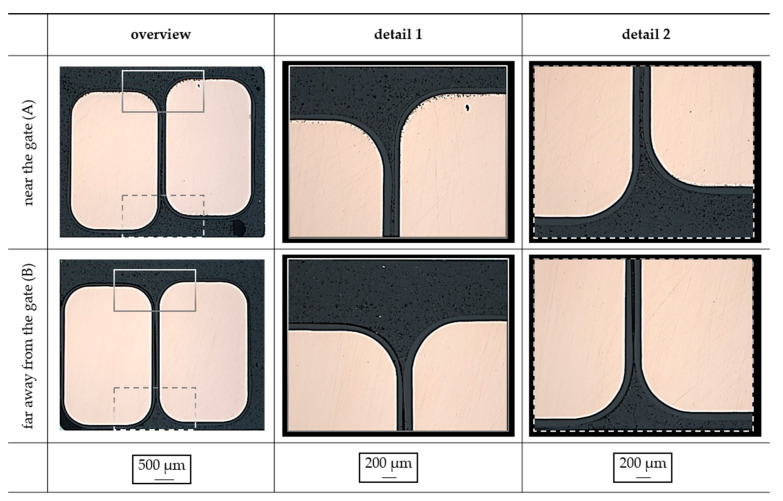
Detailed view of the conductor in the stack metal sheet to investigate the insulation of the copper wire near the gate (**A**) and far away from the gate (**B**) [exemplary: EP 3162 EMG].

The details—shown in Figure 10—reveal, that general insulation in between the two wires is possible. However, the amount of material in the gap is reduced along the flow path. With that, a general insulation of the conductor using the injection molding process is possible, but further improvement has to be made in terms of the length of the flow path to ensure proper insulation along the flow path length. So far, the two EP types did not reveal a difference in the realization of the insulation and the behavior along the flow path.

### 3.7. Average Level of Partial Discharge and Partial Discharge Inception/Extinction Voltage

**Figure 11:**
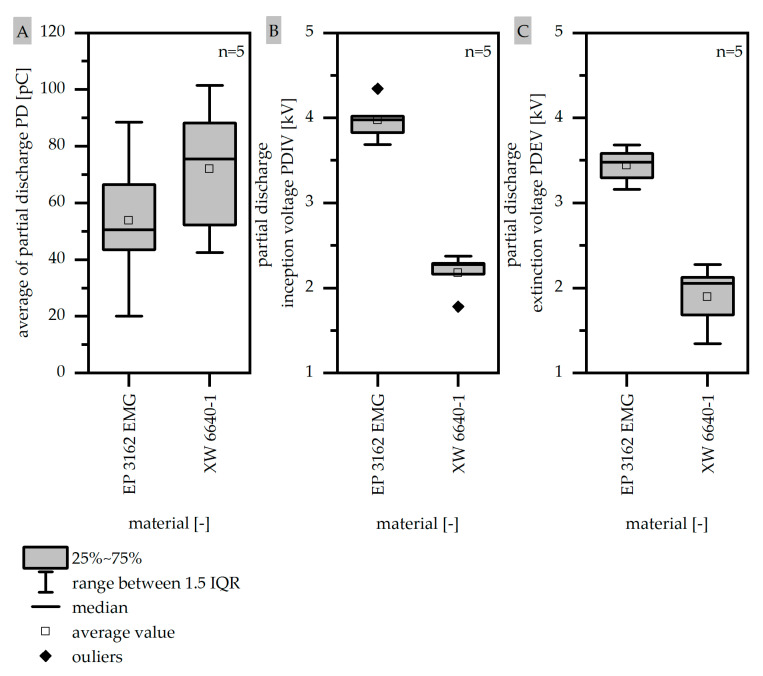
Average of partial discharge (PD) (**A**) and the partial discharge inception (PDIV) (**B**) as well as extinction voltage (PDEV) (**C**) compared for two EP types.

The electric strength of thermosets is crucial to ensure the suitability of the electrical machines during the application. To evaluate the qualification of the two EP types, the average level of partial discharge (PD) (A), the partial discharge inception voltage (PDIV) (B), and the partial discharge extinction voltage (PDEV) (C) are shown in Figure 11. The partial discharges should reach a low value to prevent electrical breakdowns due to degradation and to ensure the durability of the insulating material over the lifetime. With that, EP 3162 EMG has a slightly lower level of PD. By defining a margin of safety according to IEC60034-18-41 high PDIV and PDEV values ensure a safe operation of the electric machines without crucial PDs. In accordance with the PD-level the EP 3162 EMG has a higher value of PDIV and PDEV compared to XW 6640-1. It can be assumed, that the difference in the partial discharge resistance goes along with the change of the components within the two EP types which can have a decisive influence in electrical treeing inside the thermoset. Those two EPs probably do not only differ in terms of the filler type but further in terms of the amount of filler.

## 4. Conclusions

Based on the investigations of this paper, the suitability of epoxy based resins for the application of insulation of stators by injection molding could be determined. The main material requirements according to Table 1 can be divided into impact factors of the fabrication process and the application itself. It was shown that a general realization of insulation of stators by injection molding of epoxy based resins is possible. However, the EP type and regarding that, mainly the filler material influences the fabrication process and the properties in the application. In terms of the investigation, EP 3162 EMG reveals higher suitability in terms of the fabrication process and—in more detail—in terms of the low viscosity and the possibility of long flow paths, so far. However, the impact of the fast-curing process has to be taken into account, which might hinder long flow paths relative to the chosen process parameters. Further, EP 3162 EMG reveals advantages in the application due to a higher partial discharge resistance. However, this EP type has a deficiency regarding the thermal conductivity and the thermal linear expansion. The high suitability of XW 6640-1 in terms of the thermal conductivity and the thermal linear expansion could further be used by adopting the process. Further investigations are to be held in terms of the process parameters and the local definition of the copper wires position in the slot. It is assumed, that a general improvement of the insulation of the wires increases the suitability of XW 6640-1 in terms of the application. Further changes in the properties can be realized by different filler systems, which can—for example—even increase the thermal conductivity or the heat capacity in terms of EP 3162 EMG. The comparison of the two EP types regarding the material properties in terms of the fabrication process and the application are shown in Table 4 and reveal an intermediate result. It was also shown that the inclusion of fillers within the epoxy resin is needed to improve the thermal conductivity a. This inclusion of fillers within the material systems has an impact on the reaction kinetics and the viscosity behavior as shown.

Further investigations plan to determine the influence of the process parameters mainly in terms of insulation of the conductor and the reduction of the change of the wire position along the flow path. Moreover, a method of defined positioning of the conductor in the slot during the fabrication process needs to be found, to use the advantages of XW 6640-1 in the application. In addition, the influence of the filler system on the process conditions and the application properties will be analyzed by integrating a defined amount of fillers in pure EP material systems.

## Figures and Tables

**Table 1:** Main material requirements in terms of the fabrication process and the application for the use of thermosets to impregnate stators by injection molding.

	Material Property	Requirement
fabrication process	specific heat capacity c	high
	reaction kinetics	-
	viscosity	low
application	flow path	high
	insulation of the conductor	full and homogeneous
	thermal conductivity a	high
	thermal linear expansion ΔL	low
	average of partial discharge	low
	partial discharge inception/extinction voltage	high

**Table 2:** Specification of the material used in the investigation including density (own measurements).

Material	Manufacturer	Density ρ [g/cm^3^]
EP 3162 EMG	Raschig GmbH	1.77
XW 6640-1	Duresco GmbH	2.41

**Table 3:** Processing parameters of injection molding to fabricate test samples.

Process Parameters	Test Sample of Plate	Single Slot Sample
mass temperature [°C]	85	85
mold temperature [°C]	180	170
insert temperature [°C]	-	23
holding pressure [bar]	300	400
heating time [s]	40	40
injection speed [mm/s]	15	15

**Table 4:** Evaluation of the suitability of two EP types in terms of the main material requirements regarding the fabrication process and the application for the usage of thermosets to impregnate stators by injection molding [x: higher suitability; -: lower suitability].

	Material Property	Qualification of the Material	
		EP 3162 EMG	XW 6640-1
fabrication process	specific heat capacity c	x	-
	reaction kinetic	x	x
	viscosity	x	-
application	flow path	x	x
	insulation of the conductor	x	x
	thermal conductivity a	-	x
	thermal linear expansion ΔL	-	x
	average of partial discharge	x	-
	partial discharge inception/extinction voltage	x	-
